# Shenkang injection improves chronic kidney disease by inhibiting multiple renin-angiotensin system genes by blocking the Wnt/β-catenin signalling pathway

**DOI:** 10.3389/fphar.2022.964370

**Published:** 2022-08-17

**Authors:** Yan-Ni Wang, Hong-Jiao Liu, Li-Li Ren, Ping Suo, Liang Zou, Ya-Mei Zhang, Xiao-Yong Yu, Ying-Yong Zhao

**Affiliations:** ^1^ Faculty of Life Science and Medicine, Northwest University, Xi’an, Shaanxi, China; ^2^ Key Disciplines Team of Clinical Pharmacy, School of Food and Bioengineering, Affiliated Hospital of Chengdu University, Chengdu University, Chengdu, Sichuan, China; ^3^ Clinical Genetics Laboratory, Affiliated Hospital and Clinical Medical College of Chengdu University, Chengdu, Sichuan, China; ^4^ Department of Nephrology, Shaanxi Traditional Chinese Medicine Hospital, Xi’an, Shaanxi, China; ^5^ School of Pharmacy, Zhejiang Chinese Medical University, Hangzhou, Zhejiang, China

**Keywords:** chronic kidney disease, renal fibrosis, Shenkang injection, rhein, renin-angiotensin system, Wnt/β-catenin signalling pathway

## Abstract

Chronic kidney disease (CKD) is a major worldwide public health problem. The increase in the number of patients with CKD and end-stage kidney disease requesting renal dialysis or transplantation will progress to epidemic proportions in the next several decades. Although blocking the renin-angiotensin system (RAS) has been used as a first-line standard therapy in patients with hypertension and CKD, patients still progress towards end-stage kidney disease, which might be closely associated with compensatory renin expression subsequent to RAS blockade through a homeostatic mechanism. The Wnt/β-catenin signalling pathway is the master upstream regulator that controls multiple intrarenal RAS genes. As Wnt/β-catenin regulates multiple RAS genes, we inferred that this pathway might also be implicated in blood pressure control. Therefore, discovering new medications to synchronously target multiple RAS genes is necessary and essential for the effective treatment of patients with CKD. We hypothesized that Shenkang injection (SKI), which is widely used to treat CKD patients, might ameliorate CKD by inhibiting the activation of multiple RAS genes *via* the Wnt/β-catenin signalling pathway. To test this hypothesis, we used adenine-induced CKD rats and angiotensin II (AngII)-induced HK-2 and NRK-49F cells. Treatment with SKI inhibited renal function decline, hypertension and renal fibrosis. Mechanistically, SKI abrogated the increased protein expression of multiple RAS elements, including angiotensin-converting enzyme and angiotensin II type 1 receptor, as well as Wnt1, β-catenin and downstream target genes, including Snail1, Twist, matrix metalloproteinase-7, plasminogen activator inhibitor-1 and fibroblast-specific protein 1, in adenine-induced rats, which was verified in AngII-induced HK-2 and NRK-49F cells. Similarly, our results further indicated that treatment with rhein isolated from SKI attenuated renal function decline and epithelial-to-mesenchymal transition and repressed RAS activation and the hyperactive Wnt/β-catenin signalling pathway in both adenine-induced rats and AngII-induced HK-2 and NRK-49F cells. This study first revealed that SKI repressed epithelial-to-mesenchymal transition by synchronously targeting multiple RAS elements by blocking the hyperactive Wnt/β-catenin signalling pathway.

## Introduction

The worldwide increase in the number of chronic kidney disease (CKD) patients could be reflected in the rising number of end-stage renal disease patients who undergo renal replacement treatment, such as dialysis and transplantation (Hansrivijit et al., 2021; Kalantar-Zadeh et al., 2021; Wang et al., 2022a). CKD affects 13% of adults worldwide (Mantovani and Chiara, 2020; Rashid et al., 2022). Renal fibrosis is the common end-result of CKD (Humphreys, 2018; Bhargava et al., 2021). CKD is associated with a wide range of mechanisms, including aberrant cellular activities such as fibroblast activation, monocyte/macrophage infiltration and epithelial-to-mesenchymal transition (EMT); the activation of molecules such as renin-angiotensin system (RAS), noncoding RNAs and aryl hydrocarbon receptor (Lu et al., 2020; Zhou et al., 2021; Cao et al., 2022); and the dysregulation of pathways such as Wnt/β-catenin and transforming growth factor-β (TGF-β)/Smad signals (Wang et al., 2018a; Yu et al., 2022).

In the last several decades, experimental and clinical findings have indicated that intrarenal RAS activation plays a critical role in the pathogenesis of hypertension and CKD (Yang and Xu, 2017; Luzes et al., 2021). Considerable evidence has demonstrated that intrarenal RAS is activated by the upregulation of multiple RAS element genes, including angiotensinogen, renin, angiotensin-converting enzyme (ACE) and angiotensin II type 1 receptor (AT_1_R), after renal damage (Zhou et al., 2015a). Two important enzymes in the RAS, renin and ACE, form the principal active peptide AngII, which induces both blood pressure-dependent and blood pressure-independent renal injury. In addition to regulating blood pressure and haemodynamics, AngII activates TGF-β1 signalling pathways and directly mediates kidney fibrosis. Therefore, angiotensin-converting enzyme inhibitors (ACEIs) and angiotensin receptor blockers (ARBs) have been recommended clinically as first-line therapies in CKD patients (Chen et al., 2019a). These medications could effectively lower proteinuria and CKD progression. However, current targeted RAS treatment using ACEIs or ARBs only exhibits limited efficacy, and chronic administration of ACEIs or ARBs increases AngII and aldosterone levels, which is defined as AngII and aldosterone escape (Wang et al., 2018b). In addition, this effect might partly contribute to the compensatory upregulation of renin expression. Despite these treatments, patients with CKD still have poor outcomes. Currently, there is no effective treatment available; therefore, it is necessary to discover new effective therapies for the prevention and treatment of renal fibrosis.

The Wnt/β-catenin pathway is an evolutionarily conserved developmental signalling cascade that plays a pivotal role in regulating organ development and formation, tissue homeostasis and disease progression (Garg and Maurya, 2019; Zhu et al., 2021). Wnt/β-catenin signalling is relatively silent in the absence of Wnt ligand, and β-catenin is degraded by ubiquitin proteins after being phosphorylated by protein composites (Li et al., 2021). Under physiological conditions, β-catenin is expressed at low levels in the cytoplasm, and most of these molecules are bound to E-cadherin, serving as cell adhesion molecules (Li et al., 2021). When Wnts are activated by ligands, β-catenin is stabilized and translocates into the nucleus, and then β-catenin binds to the T-cell factor (TCF)/lymphoid enhancer-binding factor (LEF) family and forms a complex by recruiting the transcriptional coactivator cyclic adenosine monophosphate response element-binding protein-binding protein to transactivate its target genes, such as Snail1, Twist, matrix metalloproteinase-7 (MMP-7), plasminogen activator inhibitor-1 (PAI-1) and fibroblast-specific protein 1 (FSP1) (Wu et al., 2021). Activation of the Wnt/β-catenin signalling pathway is involved in various forms of CKD, such as adriamycin nephropathy, obstructive nephropathy, diabetic nephropathy (DN), polycystic kidney disease, focal glomerulosclerosis, and chronic allograft nephropathy (Zhou et al., 2015a).

Bioinformatics analysis revealed that the promoter regions of all RAS genes included putative TCF/LEF-binding sites, and β-catenin elicited the binding of LEF-1 to these sites in kidney tubular cells (Zhou et al., 2015a). The overexpression of β-catenin or Wnt ligands mediated the expression of all RAS genes. In contrast, ICG-001, a β-catenin inhibitor, ameliorated RAS induction. Transient treatment or late administration of ICG-001 mitigated the increases in proteinuria and renal damage (Zhou et al., 2015a). Treatment with ICG-001 suppressed intrarenal expression of multiple RAS genes and inhibited the expression of downstream β-catenin target genes in mice induced by adriamycin (Zhou et al., 2015a). These findings demonstrated that all RAS genes were downstream targets of the Wnt/β-catenin signalling pathway (Zuo and Liu, 2018). Inhibiting the Wnt/β-catenin signalling pathway could ameliorate renal fibrosis by synchronously inhibiting the expression of multiple RAS genes.

Many traditional Chinese medicines (TCMs) have been demonstrated to improve CKD and protect against renal fibrosis by inhibiting RAS activation and/or the Wnt/β-catenin signalling pathway (Xue et al., 2017; Chen et al., 2018a; de Souza et al., 2019). Shenkang injection (SKI) is a commonly used herbal formula that contains rhubarb (*R. tanguticum* Maxim. ex Balf. or *Rheum palmatum* L.), safflower (*Carthamus tinctorius* L.), Astragalus (*Astragalus mongholicus* Bunge), and red sage (*Salvia miltiorrhiza* Bunge). SKI quality was determined by fingerprint analysis and high-performance liquid chromatography (HPLC) as described previously (Yao et al., 2015; Xu et al., 2017). The main bioactive anthraquinones, including rhein, aloe-emodin, chrysophanol, emodin and physcion, were used for the quality control of SKI (Wang et al., 2021a). SKI can improve CKD and its complications, such as chronic nephritis, renal insufficiency, glomerulonephritis, chronic renal failure, and DN (Qin et al., 2021). With its obvious therapeutic effects and few side effects, SKI may slow the progression of CKD. Clinical results showed that 73.05% of CKD patients treated with SKI had improved renal function (Zhang et al., 2020). The final metabolite of adenine is uric acid. Excessive adenine is oxidized to 2,8-dihydroxyadenine *via* 8-hydroxyadenine by xanthine dehydrogenase. The low solubility of 2,8-dihydroxyadenine results in a mass of precipitates in renal tubules, which leads to kidney damage. Adenine leads to metabolic dysregulation resembling chronic renal insufficiency in humans. Adenine-treated rats had significantly increased hypertension (Aminzadeh et al., 2013). We hypothesized that SKI could protect kidneys through targeted inhibition of RAS-Wnt/β-catenin axis activation in CKD. To test this hypothesis, we used adenine-induced CKD rats to examine the renoprotective effects of SKI on CKD and assessed the effect of SKI on the RAS-Wnt/β-catenin signalling pathway to reveal its underlying molecular mechanisms.

## Materials and methods

### Chemicals and reagents

SKI was obtained from Shijishenkang Pharmaceutical Company Ltd. (Xi’an, Shaanxi, China). Adenine (purity ≥99.0%) was purchased from Sigma‒Aldrich Company Ltd. (St. Louis, MO, United States). Chrysophanol, emodin and rhein in SKI were isolated and identified and were purchased from Chengdu Pufei De Biotech Co., Ltd. (Chengdu, Sichuan, China). The purities of chrysophanol, emodin and rhein were 99.12%, 98.83%, and 99.25%, respectively. Primary antibodies against collagen I (ab34710, Abcam, United States), α-SMA, (ab7817, Abcam, United States), fibronectin (ab2413, Abcam, United States), E-cadherin (ab76055, Abcam, United States), ACE (sc-23908, Santa Cruz, United States), AT_1_R (ab124505, Abcam, United States), Wnt1 (ab85060, Abcam, United States), active β-catenin (05-665, Millipore, United States), β-catenin (610154, BD Transduction Laboratories, United States), Snail1 (ab180714, Abcam, United States), Twist (ab50581, Abcam, United States), MMP-7 (ab5706, Abcam, United States), PAI-1 (612024, BD Transduction Laboratories, United States) and FSP1 (ab197896, Abcam, United States) were purchased from Santa Cruz Biotechnology (Dallas, TX, United States), Proteintech Company (Wuhan, Hubei, China), Abcam Company (Cambridge, MA, United States) and BD Transduction Laboratories (New York, NJ, United States). Glyceraldehyde-3-phosphate dehydrogenase (GAPDH, 10494-1-AP) and α-tubulin (11224-1-AP) were purchased from Proteintech Company (Wuhan, Hubei, China).

### Treatment of adenine-induced chronic kidney disease rats with Shenkang injection, chrysophanol, emodin, and rhein

Six- to eight-week-old male Sprague‒Dawley rats (body weight 180–210 g) were purchased from the Central Animal Breeding House of Xi’an Jiaotong University (Xi’an, Shaanxi, China). The rats underwent an adaptation for 1 week, during which they were fed commercial feed. The rats were administered adenine by gavage as previously described (Wang et al., 2021b). The rats were divided into eight groups (*n* = 8/group): control, CKD, low-dose SKI-treated CKD, medium-dose SKI-treated CKD, high-dose SKI-treated CKD, chrysophanol-treated CKD, emodin-treated CKD and rhein-treated CKD. Except for the control group, the other groups of CKD rats were orally administered adenine (200 mg/kg/d) for 3 weeks. The treatment groups were administered SKI (10, 20, and 30 ml/kg/d), chrysophanol (30 mg/kg/d), emodin (100 mg/kg/d) or rhein (150 mg/kg/d) for 3 weeks. After 3 weeks, individual rats were placed in metabolic cages to collect 24-h urine. The rats were anaesthetized with 10% urethane, and then blood and kidneys were collected for subsequent analysis. All animal care and experimental procedures were approved by the Ethics Committee for Animal Experiments of Northwest University.

### Blood pressure measurement and renal function assessment

Systolic blood pressure (SBP) and diastolic blood pressure (DBP) were measured by rat tail plethysmography (Techman Soft, Chengdu, China). The levels of serum creatinine were determined by using an Olympus AU6402 automatic analyser.

### Cell culture

Human kidney proximal tubular epithelial cells (HK-2 cells), normal rat kidney proximal tubular epithelial cells (NRK-52E cells) and normal rat kidney interstitial fibroblast cells (NRK-49F cells) were purchased from the China Centre for Type Culture Collection. The cells were cultured in DMEM-F12 containing 4 mM L-glutamine, 1.2 g/L NaHCO_3_, 110 mg/L sodium pyruvate, 15 mM HEPES and 1,000 mg/L glucose supplemented with 10% foetal bovine serum (Gibco, Carlsbad, CA, United States) at 37°C with 5% CO_2_.

### Cell viability assay

A cell counting kit-8 (CCK-8, EnoGene, Nanjing, China) assay was used to evaluate the viability of HK-2, NRK-52E and NRK-49F cells. The cells were resuspended and seeded in 96-well plates at a density of 1×10^4^ cells/well. After 24 h, HK-2, NRK-52E and NRK-49F cells were treated with SKI (0.125, 0.25, 0.5, 1.0, 2.0, 4.0, 6.0, 8.0, 12.0, and 16.0 mg/ml) for 24 h. In addition, HK-2 cells were treated with rhein (1.0, 2.5, 5.0, 7.5, 10.0, and 12.5 μM) for 24 h. Subsequently, CCK-8 was added to each well and incubated at 37°C for 3 h. The absorbance was measured at 450 nm on a microplate reader (Thermo, New York, United States). There were six replicates for each experimental condition.

### Cell treatment with Shenkang injection and rhein

Different concentrations of SKI (0.125, 0.25, 0.5, 1.0, 2.0, and 6.0 mg/ml) or rhein (10 μM) were used to treat HK-2 cells stimulated by AngII (1.0 μM). In addition, SKI (1.0 mg/ml) or rhein (10 μM) was used to treat NRK-49F cells stimulated by AngII (1.0 μM). The angiotensin II type 1 receptor blocker losartan (LOS, 1 mM, Selleck Chemicals, Houston, United States) was used as a standard positive control.

### Light microscopy

The effects of different concentrations of SKI on the morphological changes of HK-2 cells treated with AngII (1.0 μM) were visualized with a Leica Microsystems CMS GmbH (Leica, Wetzlar, Germany) using ToupView 3.7 software.

### Immunohistochemical staining

The expression of specific proteins, including ACE, AT_1_R, Wnt1, β-catenin and FSP1, in paraffin sections of kidney tissues was performed as previously described (Miao et al., 2020).

### Immunofluorescence staining

The *in situ* expression of fibronectin, Wnt1 and β-catenin was assessed by immunofluorescence staining using an established procedure (Chen et al., 2019b). The slides were visualized with an Olympus laser-scanning confocal microscope (FV1000, Tokyo, Japan) using FV10-ASW 4.0 VIEW software.

### Western blot analysis

All solutions, tubes, and centrifuges were maintained at 0–4°C. Total proteins were extracted from the renal cortex as previously described (Choi et al., 2010). Protein levels were determined by Western blotting as previously described (Miao et al., 2020). The blots were exposed by using enhanced chemiluminescence reagent, and the levels of the target proteins were normalized to the level of GAPDH or α-tubulin. Semiquantitative analyses of specific bands were performed by using ImageJ software (version 1.48).

### Statistical analysis

The results are expressed as the mean ± SEM. Statistical analyses were performed using GraphPad Prism software (version 6.0). A two-tailed unpaired Student’s *t* test was used for comparisons between two groups. Statistically significant differences among more than two groups were analysed by one-way analysis of variance followed by Dunnett’s post-hoc tests. *p* < 0.05 was considered significant.

## Results

### Shenkang injection controlled blood pressure and inhibited intrarenal renin-angiotensin system activation in chronic kidney disease rats

**FIGURE 1:**
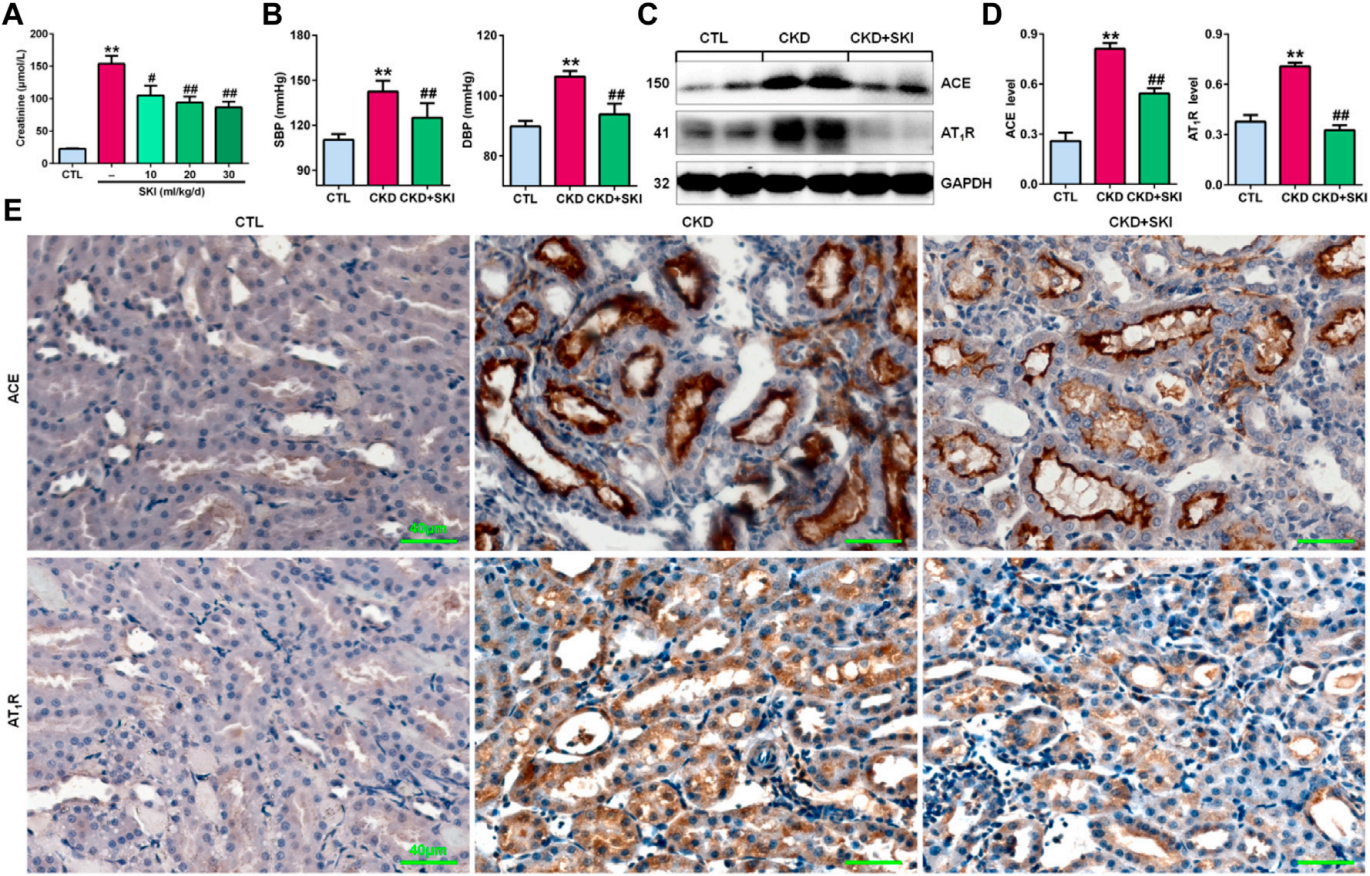
SKI controlled blood pressure and inhibited RAS activation in CKD rats. **(A)** Serum creatinine levels in adenine-induced rats treated with three doses of SKI (10, 20, 30 ml/kg/d). **(B)** SBP and DBP in adenine-induced rats treated with SKI. **(C)** Protein expression of intrarenal RAS components, including ACE and AT_1_R, in adenine-induced rats treated with SKI. **(D)** Quantitative analysis of the protein expression of intrarenal RAS components, including ACE and AT_1_R, in adenine-induced rats treated with SKI. **(E)** Immunohistochemical analysis of intrarenal ACE and AT_1_R in adenine-induced rats treated with SKI. Scale bar, 40 μm **p < 0.01 compared with CTL rats; ^#^ *p* < 0.05, ^##^ *p* < 0.01 compared with CKD rats. Abbreviations: DBP, diastolic blood pressure; SBP, systolic blood pressure; SKI, shenkang injection.

First, we examined the effects of different doses of SKI on CKD rats induced by adenine. As shown in Figure 1A, all three doses of SKI (10, 20, and 30 ml/kg/d) reduced serum creatinine levels in CKD rats, while 20 and 30 ml/kg/d SKI induced stronger effects than 10 ml/kg/d SKI. However, there were no significant differences between the effects of 20 ml/kg/d and 30 ml/kg/d SKI. Therefore, 20 ml/kg/d SKI was subsequently used for all *in vivo* experiments. Oral administration of exogenous adenine increased SBP and DBP in rats, which was accompanied by activation of the RAS, including the upregulation of intrarenal ACE and AT_1_R protein expression in adenine-induced rats (Figures 1C,D), and treatment with SKI significantly decreased blood pressure and inhibited the upregulation of ACE and AT_1_R protein expression in adenine-induced rats (Figures 1B–D). Additionally, immunohistochemistry showed that treatment with SKI significantly inhibited the upregulation of intrarenal ACE and AT_1_R protein expression in adenine-induced CKD rats (Figure 1E). Taken together, these findings demonstrated that SKI inhibited the activation of the intrarenal RAS.

### Shenkang injection suppressed the Wnt1/β-catenin signalling pathway in chronic kidney disease rats

**FIGURE 2:**
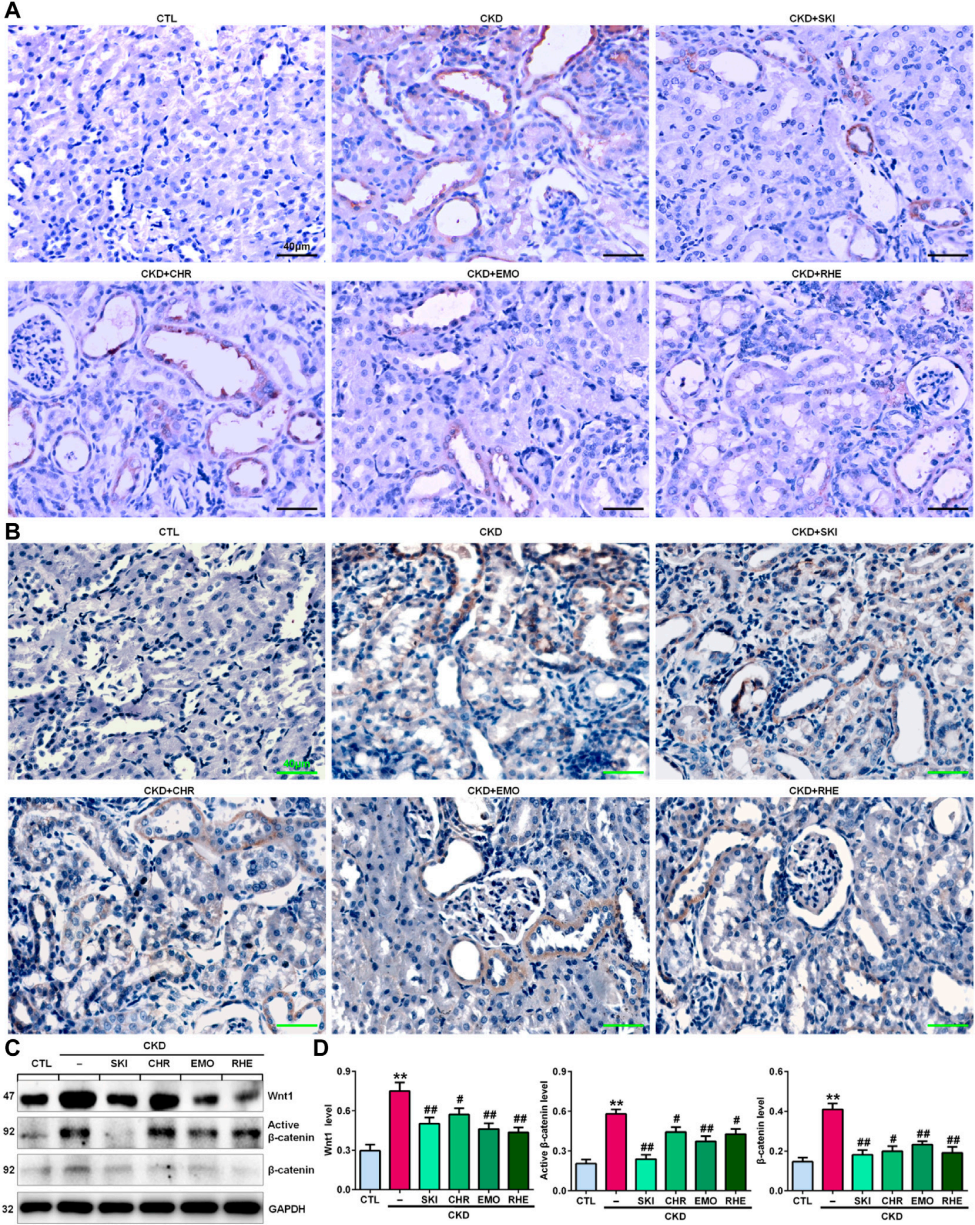
SKI suppressed Wnt1/β-catenin signalling in CKD rats. **(A)** Immunohistochemical analysis of intrarenal Wnt1 expression in the different groups. **(B)** Immunohistochemical analysis of intrarenal β-catenin expression in the different groups. **(C)** Protein expression levels of intrarenal Wnt1, active β-catenin and β-catenin in the different groups. **(D)** Quantitative analysis of the protein expression of intrarenal Wnt1, active β-catenin and β-catenin in the different groups. Scale bar, 40 μm **p < 0.01 compared with CTL rats; ^#^ *p* < 0.05, ^##^ *p* < 0.01 compared with CKD rats. Abbreviations: CHR, chrysophanol; EMO, emodin; RHE, rhein.

**FIGURE 3:**
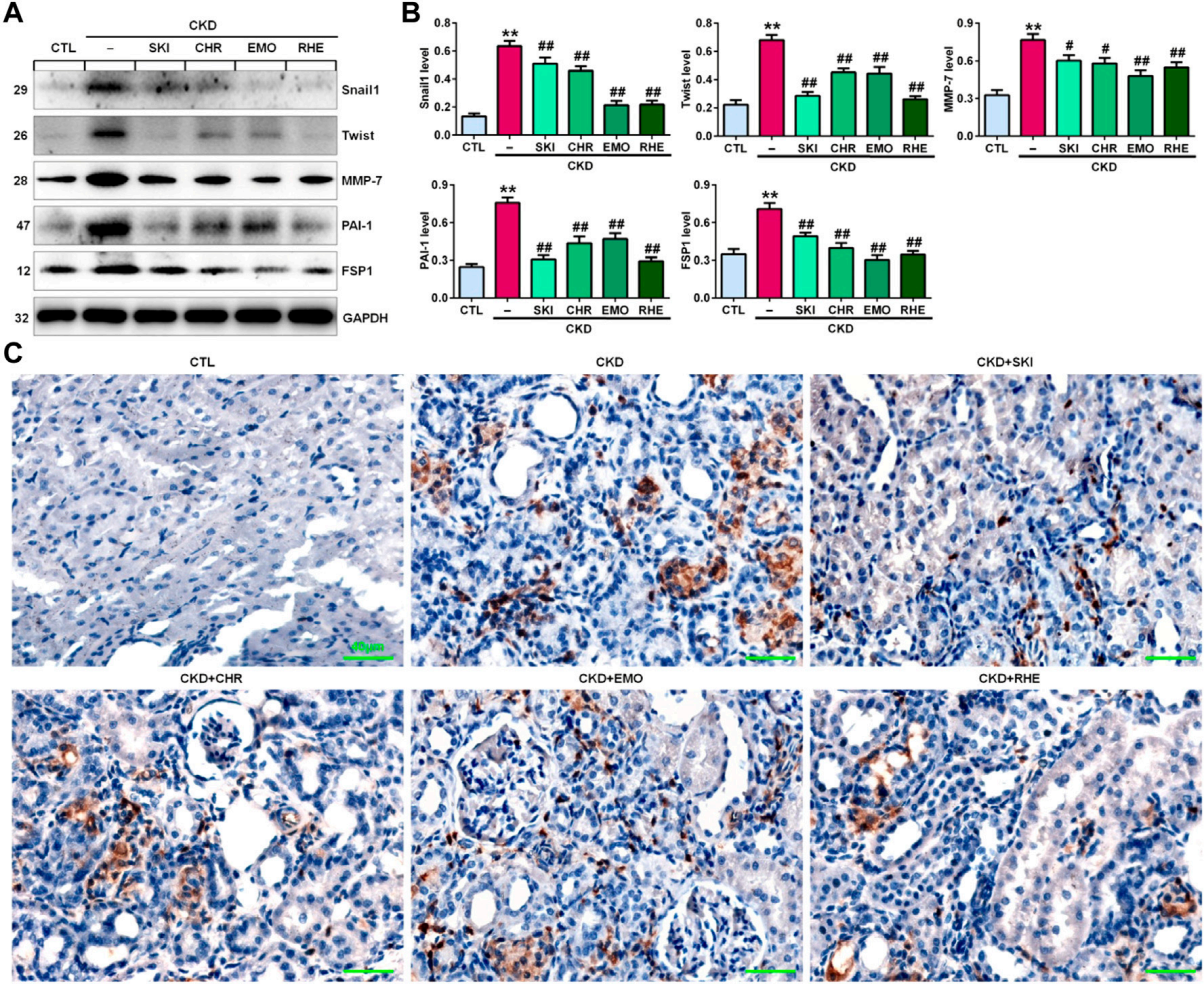
SKI inhibited the downstream target genes of β-catenin in CKD rats. **(A)** Protein expression levels of intrarenal Snail1, Twist, MMP-7, PAI-1, and FSP1 in the different groups. **(B)** Quantitative analysis of the protein expression of intrarenal Snail1, Twist, MMP-7, PAI-1, and FSP1 in the different groups. **(C)** Immunohistochemical analysis of intrarenal FSP1 expression in the different groups. Scale bar, 40 μm **p < 0.01 compared with CTL rats; ^#^ *p* < 0.05, ^##^ *p* < 0.01 compared with CKD rats.

A seminal publication indicated that all RAS genes were novel downstream targets of Wnt/β-catenin in a mouse model of nephropathy induced by adriamycin (Zhou et al., 2015a). Our previous study showed that chrysophanol, emodin, and rhein, which are anthraquinones that are major components of SKI, could ameliorate renal fibrosis in adenine-induced CKD rats (Luo et al., 2021). We further examined whether SKI and these three anthraquinones had inhibitory effects on the activation of the Wnt1/β-catenin signalling pathway in adenine-induced CKD rats. Immunohistochemical analysis showed that adenine upregulated intrarenal Wnt1 protein expression (Figure 2A), which was accompanied by the upregulation of intrarenal β-catenin protein expression in CKD rats (Figure 2B). However, treatment with SKI and the three anthraquinones significantly inhibited the upregulation of intrarenal Wnt1 and β-catenin protein expression in CKD rats (Figures 2A,B). Similarly, upregulated protein expression of intrarenal Wnt1 and β-catenin was observed in CKD rats by Western blotting, while treatment with SKI and the three anthraquinones significantly inhibited the upregulation of these intrarenal proteins in adenine-induced CKD rats (Figures 2C,D). The upregulation of intrarenal β-catenin protein expression was accompanied by the upregulation of downstream β-catenin proteins, including Snail1, Twist, MMP-7, PAI-1, and FSP1, in CKD rats, while treatment with SKI and the three anthraquinones significantly inhibited this upregulation of protein expression in CKD rats (Figures 3A,B). Additionally, immunohistochemical analysis showed that treatment with SKI and the three anthraquinones significantly inhibited intrarenal FSP1 expression in CKD rats (Figure 3C). Intriguingly, rhein exerted the strongest inhibitory effect on the Wnt1/β-catenin signalling pathway among the three anthraquinones. Therefore, rhein was selected for further bioactivity evaluation *in vitro*. Overall, these findings demonstrated that SKI inhibited activation of the intrarenal Wnt1/β-catenin signalling pathway.

### Effects of Shenkang injection on cell viability

**FIGURE 4:**
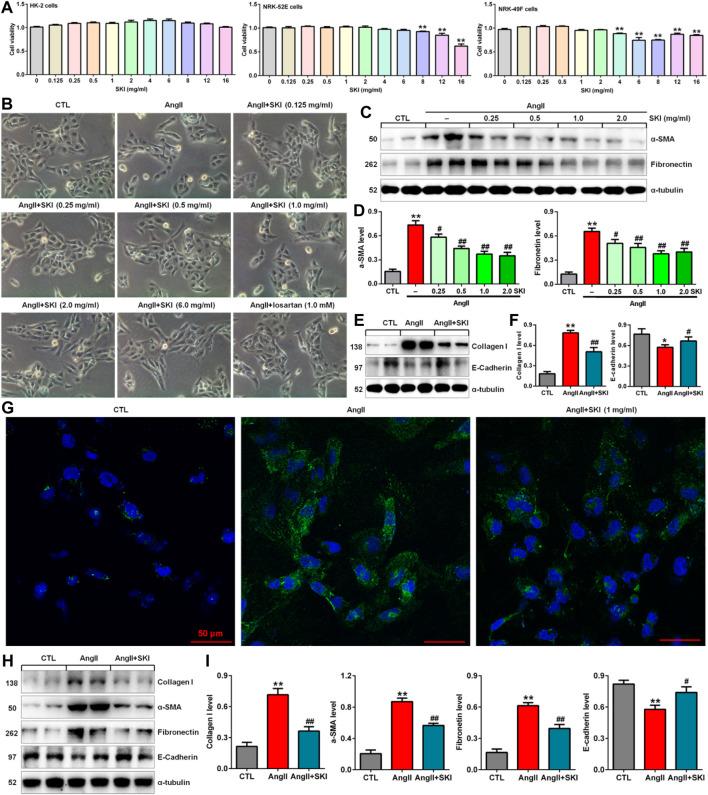
SKI improved morphological changes and injury in AngII-induced HK-2 cells. **(A)** Cell viability analysis after HK-2, NRK-52E and NRK-49F cells were treated with increasing concentrations of SKI (0–16 mg/ml). **(B)** Morphological observation in AngII-induced HK-2 cells treated with different concentrations of SKI (0–6.0 mg/ml) and losartan (1.0 mM) at 48 h. **(C)** Protein expression of α-SMA and fibronectin in AngII-induced HK-2 cells treated with different concentrations of SKI (0.25, 0.5, 1.0, 2.0 mg/ml). **(D)** Quantitative analysis of the protein expression of α-SMA and fibronectin in AngII-induced HK-2 cells treated with different concentrations of SKI (0.25, 0.5, 1.0, 2.0 mg/ml) for 48 h. **(E)** Protein expression of collagen I and E-cadherin in AngII-induced HK-2 cells treated with SKI. **(F)** Quantitative analysis of the protein expression of collagen I and E-cadherin in AngII-induced HK-2 cells treated with SKI. **(G)** Representative immunofluorescent analysis of fibronectin in AngII-induced HK-2 cells treated with SKI. **(H)** Protein expression of collagen I, α-SMA, E-cadherin and fibronectin in AngII-induced NRK-49F cells treated with SKI. **(I)** Quantitative analysis of the protein expression of collagen I, α-SMA, E-cadherin and fibronectin in AngII-induced NRK-49F cells treated with SKI. *p < 0.05, **p < 0.01 compared with the control group; ^#^ *p* < 0.05, ^##^ *p* < 0.01 compared with the AngII-induced group.

To investigate the toxicity of SKI to cells, we cultured HK-2, NRK-52E and NRK-49F cells with SKI (0.125–16.0 mg/ml) and examined cell viability. As shown in Figure 4A, 0.125–16.0 mg/ml, 0.125–6.0 mg/ml and 0.125–2.0 mg/ml SKI had no significant effects on the viability of HK-2, NRK-52E and NRK-49F cells, respectively, but 8.0 mg/ml SKI 4.0 mg/ml SKI significantly reduced the viability of NRK-52E cells and NRK-49F cells, respectively. Therefore, SKI concentrations between 0.125 and 6.0 mg/ml were used to assess the effect of SKI on morphological changes in HK-2 cells stimulated with AngII.

### Shenkang injection inhibited AngII-induced morphological changes and injury in intrarenal cells

High blood pressure led to RAS activation in adenine-induced rats. Therefore, we used AngII stimulation to investigate the effect of SKI on HK-2 cells. As shown in Figure 4B, HK-2 cells in the control group exhibited a pebble-like appearance, while the cells showed a decrease in cell–cell contacts and became a more elongated following stimulation with AngII (1.0 μM) for 48 h. Treatment with SKI could improve renal cell injury. Notably, treatment with SKI (0.25–2.0 mg/ml) significantly ameliorated AngII-induced morphological changes in HK-2 cells. We further determined the effect of SKI on profibrotic proteins such as α-SMA and fibronectin in AngII-induced HK-2 cells. Treatment with SKI exhibited a concentration-dependent inhibitory effect on the protein expression of α-SMA and fibronectin in HK-2 cells stimulated by AngII (Figures 4C,D). Of note, 1.0 mg/ml SKI showed a stronger inhibitory effect on profibrotic protein expression than the other concentrations of SKI. Collectively, 1.0 mg/ml SKI was selected to analyse the molecular mechanism by which SKI protects against renal fibrosis *in vitro*.

Our results further showed that treatment with SKI significantly inhibited the protein expression of collagen I and E-cadherin in AngII-induced HK-2 cells (Figures 4E,F). Additionally, immunofluorescent analysis showed that treatment with SKI (1.0 mg/ml) significantly inhibited the protein expression of fibronectin in HK-2 cells stimulated by AngII (Figure 4G). Similarly, treatment with SKI significantly inhibited the protein expression of collagen I, α-SMA, fibronectin and E-cadherin in AngII-induced NRK-49F cells (Figures 4H,I). These results demonstrated that SKI could inhibit EMT in from HK-2 and NRK-49F cells. Collectively, our findings demonstrated that SKI exerted a significant inhibitory effect on profibrotic protein expression.

### Shenkang injection inhibited the renin-angiotensin system/Wnt/β-catenin signalling axis in AngII-induced HK-2 cells

**FIGURE 5:**
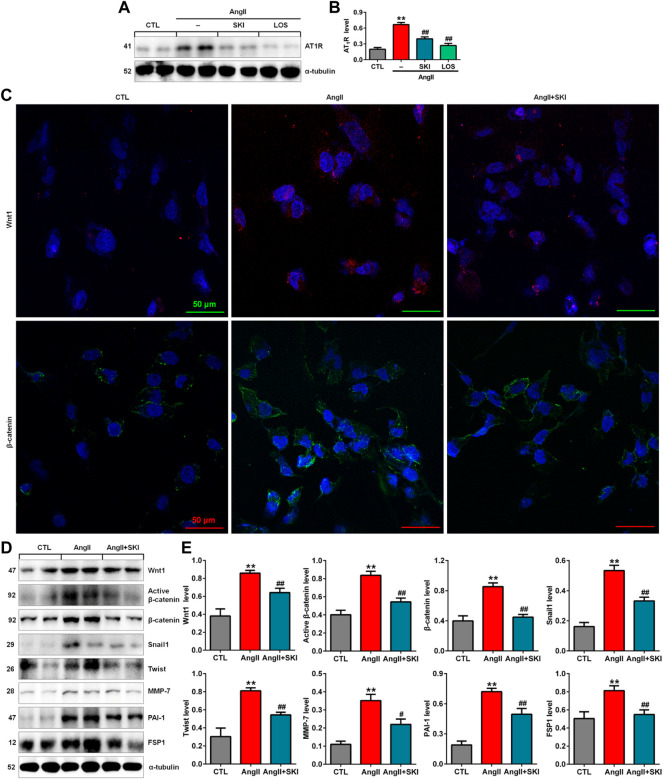
SKI inhibited the RAS/Wnt/β-catenin signalling axis in AngII-induced HK-2 cells. **(A)** Protein expression of AT_1_R in AngII-induced HK-2 cells treated with SKI and losartan. **(B)** Quantitative analysis of the protein expression of AT_1_R in AngII-induced HK-2 cells treated with SKI and losartan. **(C)** Representative immunofluorescent analysis of Wnt1 and β-catenin in AngII-induced HK-2 cells treated with SKI. **(D)** Protein expression of Wnt1, active β-catenin and β-catenin, as well as its downstream target gene products, including Snail1, Twist, MMP-7, PAI-1, and FSP1, in AngII-induced HK-2 cells treated with SKI. **(E)** Quantitative analysis of Wnt/β-catenin signalling pathway protein expression in AngII-induced HK-2 cells treated with SKI. **p < 0.01 compared with the control group; ^#^ *p* < 0.05, ^##^ *p* < 0.01 compared with the AngII-induced group.

Our *in vivo* experiment demonstrated that SKI inhibited intrarenal RAS activation in adenine-induced rats. We further investigated the effect of SKI on the RAS in AngII-induced HK-2 cells. The upregulation of AT1R protein expression was observed in AngII-induced HK-2 cells, while SKI significantly abrogated the increase in AT_1_R protein expression, which was consistent with the inhibitory effect of losartan (Figures 5A,B). A previous study demonstrated that all RAS components were downstream targets of Wnt/β-catenin signalling. Immunofluorescence analysis showed that treatment with SKI significantly inhibited the protein expression of Wnt1 and β-catenin in HK-2 cells stimulated by AngII, and treatment with SKI significantly inhibited the expression of these two proteins in AngII-treated HK-2 cells (Figure 5C). Western blot analysis showed that the protein expression of Wnt1, active β-catenin and β-catenin was upregulated in AngII-induced HK-2 cells, which was accompanied by upregulated expression of downstream β-catenin targets, including Snail1, Twist, MMP-7, PAI-1. and FSP1, while treatment with SKI significantly inhibited this upregulation in protein expression in AngII-induced HK-2 cells (Figures 5D,E). These results demonstrate that SKI exerted its renoprotective effect by inhibiting the RAS/Wnt/β-catenin signalling axis.

### Rhein inhibited epithelial-to-mesenchymal transition by blocking renin-angiotensin system activation in AngII-induced HK-2 cells

**FIGURE 6:**
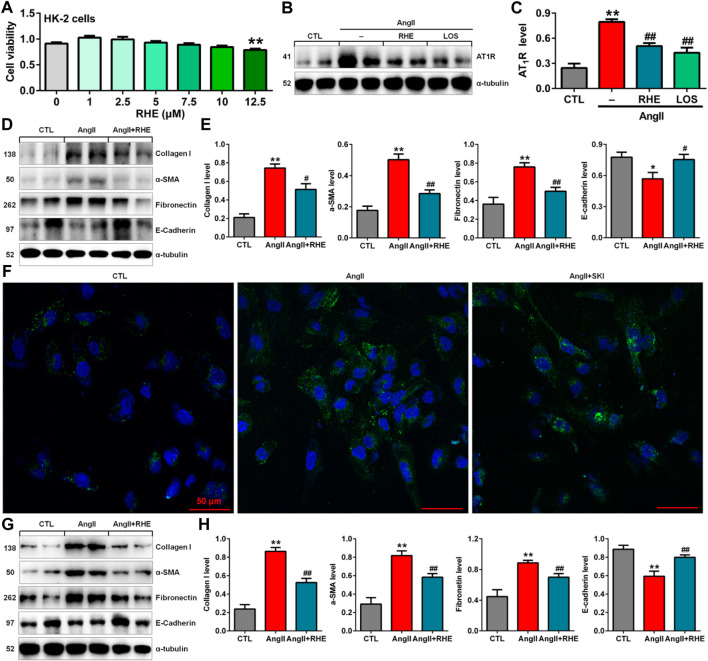
Rhein inhibited EMT by blocking the RAS in AngII-induced HK-2 cells. **(A)** Cell viability analysis after HK-2 cells were treated with increasing concentrations of rhein (0–12.5 μM). **(B)** Protein expression of AT_1_R in AngII-induced HK-2 cells treated with rhein and losartan. **(C)** Quantitative analysis of the protein expression of AT_1_R in AngII-induced HK-2 cells treated with rhein and losartan. **(D)** Protein expression of collagen I, α-SMA, E-cadherin and fibronectin in AngII-induced HK-2 cells treated with rhein. **(E)** Quantitative analysis of the protein expression of collagen I, α-SMA, E-cadherin and fibronectin in AngII-induced NRK-49F cells treated with rhein. **(F)** Representative immunofluorescent analysis of fibronectin in AngII-induced HK-2 cells treated with SKI. **(G)** Protein expression of collagen I, α-SMA, E-cadherin and fibronectin in AngII-induced NRK-49F cells treated with rhein. **(H)** Quantitative analysis of the protein expression of collagen I, α-SMA, E-cadherin and fibronectin in AngII-induced NRK-49F cells treated with rhein. *p < 0.05, **p < 0.01 compared with the control group; ^#^ *p* < 0.05, ^##^ *p* < 0.01 compared with the AngII-induced group.

The previous *in vivo* experiments demonstrated that rhein exerted the strongest inhibitory effects on renal fibrosis and the Wnt/β-catenin signalling pathway among the three anthraquinones. Therefore, the effect of rhein on AngII-induced HK-2 cells was further investigated. Treatment with 1.0–10.0 μM rhein for at 24 h had no significant effect on HK-2 cell viability, while 12.5 μM rhein significantly reduced cell viability (Figure 6A). Therefore, 10 μM rhein was ultimately selected to examine the molecular mechanism *in vitro*.

Treatment with rhein significantly inhibited the increase in AT_1_R protein expression, which was consistent with the inhibitory effect of losartan (Figures 6B,C). Treatment with rhein significantly inhibited the protein expression of collagen I, α-SMA, fibronectin and E-cadherin AngII-induced HK-2 cells (Figures 6D,E). Additionally, immunofluorescent analysis showed that treatment with rhein significantly inhibited the protein expression of fibronectin in HK-2 cells stimulated by AngII (Figure 6F). Similarly, treatment with rhein significantly inhibited the protein expression of collagen I, α-SMA, fibronectin and E-cadherin in AngII-induced NRK-49F cells (Figures 6G,H). These results indicated that rhein could inhibit EMT.

### Rhein inhibited the Wnt/β-catenin signalling pathway in AngII-induced HK-2 cells

**FIGURE 7:**
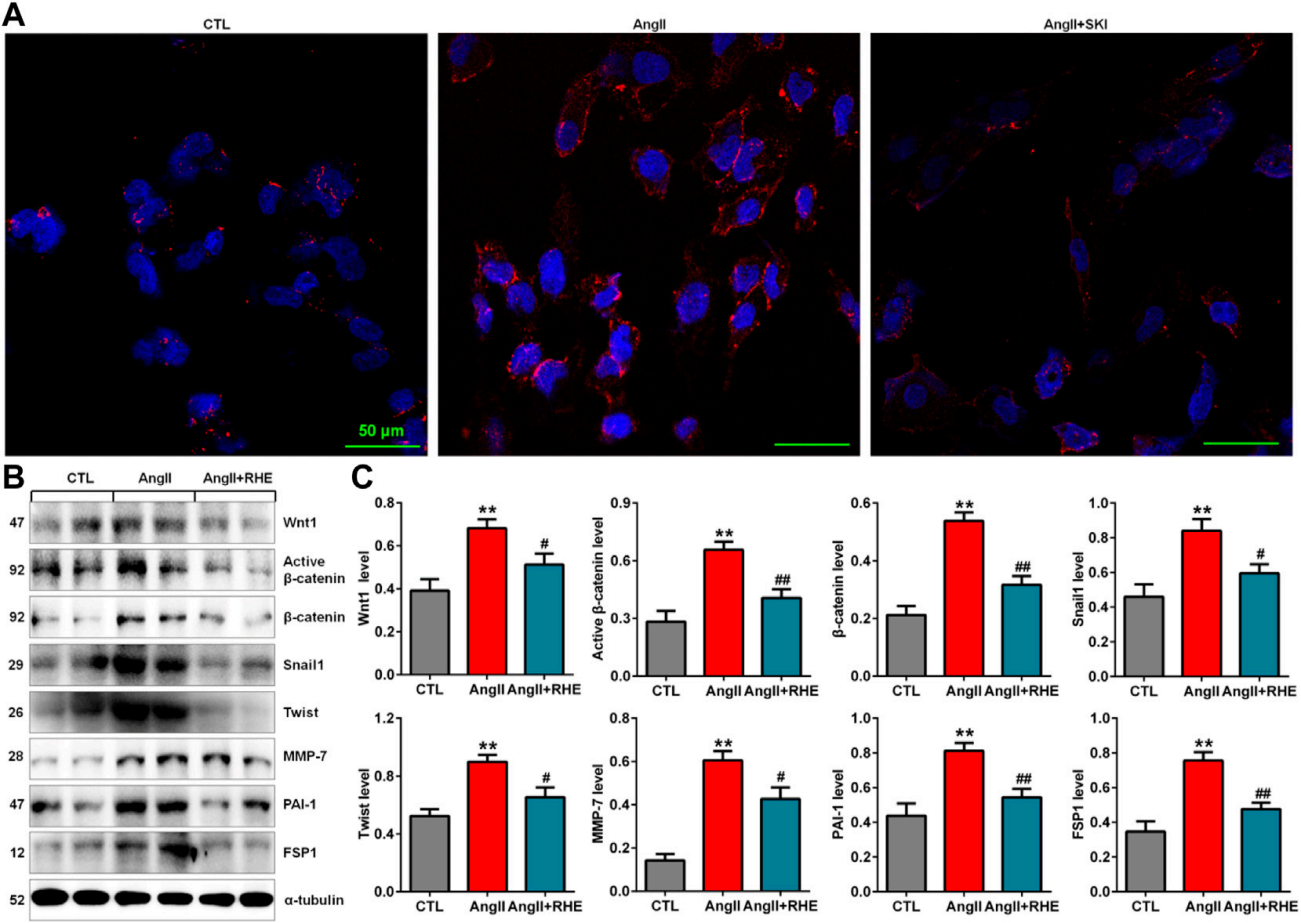
Rhein inhibited the Wnt/β-catenin signalling pathway in AngII-induced HK-2 cells. **(A)** Representative immunofluorescent analysis of β-catenin in AngII-induced HK-2 cells treated with rhein. **(B)** Protein expression of Wnt1, active β-catenin and β-catenin, as well as its downstream target gene products, including Snail1, Twist, MMP-7, PAI-1, and FSP1, in AngII-induced HK-2 cells treated with rhein. **(C)** Quantitative analysis of the protein expression of Wnt/β-catenin signalling pathway factors in AngII-induced HK-2 cells treated with rhein. **p < 0.01 compared with the control group; ^#^ *p* < 0.05, ^##^ *p* < 0.01 compared with the AngII-induced group.

Immunofluorescence analysis showed that treatment with rhein significantly inhibited the protein expression of β-catenin in HK-2 cells stimulated with AngII (Figure 7A). Western blot analysis also showed that treatment with rhein significantly inhibited the upregulation of Wnt1, active β-catenin and β-catenin protein expression in AngII-induced HK-2 cells, which was accompanied by inhibition of the downstream β-catenin targets including Snail1, Twist, MMP-7, PAI-1, and FSP1 (Figures 7B,C). These results demonstrated that rhein exerted its renoprotective effect by inhibiting the Wnt/β-catenin signalling pathway.

## Discussion

The current study demonstrates that SKI and its three components chrysophanol, emodin, and rhein inhibit tubulointerstitial fibrosis by inhibiting the RAS/Wnt/β-catenin signalling axis in adenine-induced CKD rats. Recent studies show that emodin ameliorates tubulointerstitial fibrosis (Wang et al., 2022b; Xu et al., 2022). In addition, several publications revealed that rhein improves renal fibrosis and is associated with the nuclear factor kappa B and Twist1 pathways (Chen et al., 2022; Song et al., 2022). Many studies have demonstrated that RAS activation is closely associated with various diseases (Bakhle, 2020; Bhullar et al., 2021; Lizaraso-Soto et al., 2021; Mikusic et al., 2021; Walther et al., 2021). The presence of a local or tissue-based RAS has been well documented and is considered to be a pivotal player in the pathogenesis of CKD and its complications (Yang and Xu, 2017). The kidneys express all RAS elements, and intrarenal AngII production not only regulates glomerular haemodynamics and tubular sodium transport but also mediates many inflammatory and fibrotic pathways (Kobori et al., 2007). Over the past several decades, blood pressure control and RAS blockade have been considered cornerstones of preventing CKD or slowing disease progression, following key background and clinical evidence on the correlation among hypertension and activation of RAS with kidney damage (Kobori et al., 2007; Hsu and Tain, 2021). On the one hand, increased blood pressure is a major risk factor for CKD, and on the other hand, renal damage can lead to hypertension (Kobori et al., 2007). The published findings indicated a strong relationship between hypertension and the risk of renal functional decline or ESRD (Kobori et al., 2007). Therefore, numerous studies have suggested that ACEIs and ARBs are recommended as first-line therapies for hypertension in CKD patients (Hsu and Tain, 2021). Although ACEIs and ARBs can ameliorate CKD progression, the results are often unsatisfactory, and many patients progress to ESRD or even die. One of the important potential reasons is compensatory renin levels followed by RAS inhibition through a homeostatic mechanism (Zhang et al., 2008). Increased renin expression mediates the profibrotic effect though an angiotensin-independent mechanism involving binding to the prorenin/renin receptor (Nguyen et al., 2002). The active renin inhibitor aliskiren was shown to effectively treat hypertension and CKD (Jhund et al., 2015; Zuo and Liu, 2018). However, aliskiren did not show significant beneficial effects on renal and cardiovascular diseases compared with ARB alone but markedly increased the risk of side effects based on the ONTARGET and aliskiren in left ventricular hypertrophy trials (Mann et al., 2008). Furthermore, ACEIs and ARBs repressed the activity of RAS elements but did not inhibit their expression. Therefore, there is still a clear need for additional strategies to synchronously block multiple RAS genes more effectively to reduce the progression of CKD.

A seminal publication revealed that multiple RAS genes were direct downstream targets of the Wnt/β-catenin signalling pathway (Zhou et al., 2015a). After being activated, β-catenin translocated to the nucleus, where it bound to and activated transcription factors in the TCF and LEF families to mediate the transcription of target genes. The common TCF/LEF binding sequence was (A/T)(A/T)CAA(A/T)G. The bioinformatics results indicated the presence of presumptive TCF/LEF-binding sites in promoter areas of RAS genes, including AGT, renin, ACE, AT_1_R, and AT_2_R (Zhou et al., 2015a). An *in vitro* study showed that increased β-catenin expression promoted the binding of LEF-1 to sites in these RAS genes, and the activation of β-catenin or many Wnt ligands could induce the expression of AGT, renin, ACE, and AT_1_R (Zhou et al., 2015a). Collectively, these findings indicated that canonical Wnt/β-catenin signalling was a master upstream mediator that could synchronously modulate the expression of these RAS genes in diseased kidneys.

The β-catenin inhibitor ICG-001 effectively inhibited the upregulation of four RAS elements in kidneys. Therefore, mechanism of the inhibitory effect of ICG-001 was fully different from that of RAS intervention therapy. Although ACEIs or ARBs target one specific element of the RAS, ICG-001 inhibited the upregulation of all RAS genes. Further analysis indicated that ICG-001 could lower blood pressure (Xiao et al., 2019). ICG-001 treatment could lower AngII-induced hypertension through the Wnt/β-catenin signalling pathway. Interestingly, chronic AngII infusion could trigger the expression of multiple Wnt genes in rats and rat kidney interstitial fibroblasts (NRK-49F), thereby forming a vicious cycle between RAS and Wnt/β-catenin (Xiao et al., 2019). In 5/6 nephrectomised rats, intrarenal β-catenin expression was significantly increased, and ICG-001 treatment could inhibit the increase in blood pressure and the protein expression of AGT, renin, ACE, and AT_1_R. Moreover, ICG-001 treatment could reduce the levels of albuminuria, serum creatinine and urea, inhibit intrarenal protein expression of PAI-1, collagen I and fibronectin and repress the infiltration of inflammatory cells, including CD3^+^ T cells and CD68^+^ monocytes/macrophages (Xiao et al., 2019). In human kidney proximal tubular epithelial cells (HKC-8), losartan could inhibit Wnt/β-catenin-induced expression of α-SMA, Snail1 and fibronectin, indicating that the expression of fibrotic genes associated with Wnt/β-catenin signalling was dependent on RAS activation (Xiao et al., 2019). In addition, the antiaging protein Klotho, which is highly expressed in the tubular epithelium of normal adult kidneys, could bind to and functionally sequester Wnt ligands; therefore, Klotho is an endogenous Wnt antagonist that inhibits Wnt/β-catenin signalling activity (Zhou et al., 2013). Exogenous Klotho expression suppressed the upregulation of AGT, renin, ACE, and AT_1_R protein expression and normalized blood pressure in 5/6 nephrectomised rats and unilateral ureteral obstruction (UUO) mice (Zhou et al., 2015b). Klotho can also repress β-catenin expression and ameliorate renal fibrosis (Zhou et al., 2013). Collectively, these findings uncovered a mechanistic link between the Wnt/β-catenin signalling pathway and blood pressure regulation. Hyperactivity of the Wnt/β-catenin signalling pathway could promote hypertension and renal injury through RAS activation.

A growing body of evidence suggested that inhibiting RAS activation by a number of TCMs, including TCM formulas, single herbs and identified compounds, could slow CKD and renal fibrosis (Yang et al., 2019). For example, Zhen-wu-tang is a classic TCM formula that is specifically used to treat CKD. Treatment with Zhen-wu-tang could blunt hyperglycaemia-induced intrarenal AngII levels in rats with DN induced by streptozotocin (Cai et al., 2010). Yiqi Huaju formula could lower arterial pressure and downregulate the mRNA expression of intrarenal renin, ACE and AT_1_R in rats with salt-sensitive hypertension induced by a high-salt and high-fat diet, but this formula did not affect the activities of plasma renin, ACE or AngII (He et al., 2015). Wulingsan has long been used to regulate body fluid homeostasis. Treatment with Wulingsan could lower adriamycin-induced intrarenal angII levels in rats with nephrotic syndrome. In addition, treatment with Wulingsan could lower plasma renin activity and aldosterone levels in rats (Ahn et al., 2012). Two previous studies have demonstrated the effect of SKI components, including *Astragali Radix* and *C. tinctorius,* on RAS elements, such as ACE, AngII, and AT_1_R, in renal injury rats. A previous study indicated that total flavonoids of *A. Radix*, one of the major components of SKI, could inhibit intrarenal ACE mRNA expression in rats with adriamycin-induced nephropathy (Zhang et al., 2018). Another study indicated that the ethanol extract of *C. tinctorius*, one of the major components of SKI, could inhibit serum ACE activity, plasma AngII levels, and aortic AT_1_R protein expression in 2K-1C hypertensive rats (Bunbupha et al., 2018). Treatment with *Nigella sativa* extract repressed AngII expression in rats with UUO (Hosseinian et al., 2018). 2,3,5,4′-Tetrahydroxystilbene-2-O-β-d-glucoside is one of the important components of *Polygonum multiflorum* Thunb. Treatment with 2,3,5,4′-tetrahydroxystilbene-2-O-β-d-glucoside could inhibit hyperglycaemia-induced mRNA and protein expression of AGT, renin, ACE, and AT_1_R in mice with DN induced by streptozotocin (Chen et al., 2016). Collectively, these findings suggest that TCM protects against renal injury by modulating RAS activation.

Although RAS activation is associated with various pathways, the most compelling evidence highlighted that the Wnt/β-catenin signalling pathway was the master upstream regulator that controlled the expression of all RAS elements (Zhou et al., 2015a), suggesting that targeting this upstream factor might be an effective strategy to prevent and treat CKD in patients with hypertension. Our latest review showed that targeting the Wnt/β-catenin signalling pathway was a viable therapeutic strategy to treat renal fibrosis (Liu et al., 2019; Li et al., 2021). Based on clinical observations and experimental studies, several TCM formulas have been demonstrated to inhibit the Wnt/β-catenin signalling pathway in renal injury. Clinically, a randomized controlled trial showed that treatment with Qingshen granules could lower the levels of serum Wnt1, β-catenin, α-SMA, and E-cadherin in patients with chronic renal failure (Wang et al., 2019). In cell- and rat/mouse-based models, several studies have demonstrated the effect of TCM formulas on the Wnt/β-catenin signalling pathway in renal injury. For example, Huang Gan formula, a new TCM compound, could lower serum creatinine and urea levels, inhibit oxidative stress and ameliorate tubulointerstitial fibrosis in rats with adenine-induced CKD (Mo et al., 2015). Treatment with the Huang Gan formula improved renal function by decreasing the levels of serum creatinine and urea, as well as urine protein, and increasing the creatinine clearance rate in 5/6 nephrectomised rats. Huang Gan formula could modulate the Wnt/β-catenin signalling pathway by inhibiting the expression of Wnt1, β-catenin, TCF4 and fibronectin (Mo et al., 2015). In addition, Mahuang Fuzi and Shenzhuo decoction inhibited β-catenin expression and phosphorylation in high glucose-induced podocytes (Dai et al., 2020). Moreover, Huayu Tongluo herbs could reduce proteinuria by inhibiting the protein expression of Wnt4, glycogen synthase kinase-3 beta (GSK3β), phosphorylated GSK3β and β-catenin in diabetic rats induced by streptozotocin (Bai et al., 2017). A previous study indicated that root water and ethanol extracts, as well as stem and leaf water and ethanol extracts of *S. miltiorrhiza*, one of the major components of SKI, could repress the protein expression of Wnt4 and β-catenin in DN rats induced by a high-fat diet and streptozotocin (Xiang et al., 2019). Although these findings indicated that activation of the Wnt/β-catenin signalling pathway was involved in the inhibitory effect of TCM formulas on renal fibrosis, no publications have demonstrated that TCM formulas protect against renal fibrosis by modulating both the hyperactive Wnt/β-catenin signalling pathway and RAS activation. Our current study was the first to demonstrate that SKI could lower hypertension and abolish RAS activation by inhibiting the hyperactive Wnt/β-catenin signalling pathway.

In contrast to TCM formulas, our previous studies have indicated that a number of isolated compounds from diuretic TCMs, such as *Poria cocos*, *Alismatis rhizome*, and *Polyporus umbellatus,* could abolish RAS activation by inhibiting the hyperactive Wnt/β-catenin signalling pathway (Chen et al., 2018a). *P. cocos* (Schw.) Wolf (Polyporaceae), a well-known edible mushroom, is typically used in functional foods, nutraceuticals, dietary supplements and medications. *P. cocos* exerted various pharmacological effects, such as anti-inflammatory, antioxidant, diuretic and antifibrotic effects. We have isolated and identified a number of novel tetracyclic triterpenoids, such as poricoic acid ZC, poricoic acid ZD, poricoic acid ZE, poricoic acid ZG, poricoic acid ZH, poricoic acid ZI, poricoic acid ZM and poricoic acid ZP, from *P. cocos* (Wang et al., 2018a; Wang et al., 2018b; Chen et al., 2019c; Wang M. et al., 2020). Further pharmacological experiments have demonstrated that poricoic acid ZC, poricoic acid, ZD poricoic acid ZG, and poricoic acid ZH exhibit robust inhibitory effects on the protein expression of all RAS elements, including AGT, renin, ACE, and AT_1_R, in TGF-β1- and AngII-induced HK-2 cells and/or UUO mice. Treatment with these compounds could also inhibit the protein expression of Wnt1 and β-catenin, as well as downstream target genes, including Snail1, Twist, MMP-7, PAI-1, and FSP1 (Wang et al., 2018a; Wang et al., 2018b). In addition, our studies demonstrated that poricoic acid ZE showed a strong inhibitory effect on renin compared with poricoic acid ZC and poricoic acid ZD in TGF-β1- and AngII-induced HK-2 cells and UUO mice (Wang et al., 2018a). Similarly, *A. rhizome*, a diuretic TCM, exerts renoprotective effects (Tian et al., 2014). 25-O-Methylalisol F was isolated and identified from *A. rhizome* and could ameliorate renal injury by repressing multiple RAS elements by inhibiting the hyperactive Wnt/β-catenin signalling pathway (Chen et al., 2018b). In addition, our previous *in vitro* experiment indicated that pachymic acid B from *P. cocos*, alisol B 23-acetate from *A. rhizome* and ergone from *P. umbellatus* could inhibit the protein expression of Snail1, Twist, MMP-7, PAI-1, and FSP1 (Chen et al., 2017). In addition, our recent studies showed that poricoic acid A, as a modulator of tryptophan hydroxylase-1 expression, inhibited renal fibrosis by regulating β-catenin protein stability and induced transcription (Chen et al., 2020). Increased evidence has indicated that acute kidney injury (AKI) is one of the major risk factors for renal fibrosis progression (Xiao et al., 2016). Our previous studies showed that poricoic acid A could block AKI-to-CKD progression and renal fibrosis by modulating the Wnt/β-catenin pathway in renal ischaemia‒reperfusion injury and hypoxia/reoxygenation- or TGF-β1-induced HK-2 cells (Chen et al., 2019d). Taken together, these findings indicated that these diuretic TCM-derived components could abolish kidney damage by inhibiting multiple RAS elements *via* the Wnt/β-catenin signalling pathway. In addition, several publications have demonstrated the inhibitory effects of isolated compounds on the hyperactive Wnt/β-catenin signalling pathway in diabetes-induced renal injury. For example, rhein is an anthraquinone that is extracted from rhubarb, which is one of the major components of SKI. Treatment with rhein inhibited the upregulation of Wnt1 protein expression and the phosphorylation of β-catenin and GSK3β in db/db mice with DN (Duan et al., 2016). *In vitro* experiments from the same research group further revealed that treatment with rhein could inhibit the upregulation of Wnt3a, β-catenin and phosphorylated GSK3β protein expression in high glucose-induced podocytes (Duan et al., 2017). Astragaloside IV is a saponin extracted from *A. mongholicus*, which is another herb in SKI. Treatment with astragaloside IV inhibited the protein expression of Wnt1 and β-catenin in DN rats induced by a high-fat diet and streptozotocin (Wang et al., 2020b). Similarly, treatment with *Tripterygium wilfordii* significantly inhibited the hyperglycaemia-induced expression of Wnt1 and β-catenin at both the mRNA and protein levels in diabetic rats induced by streptozotocin (Chang et al., 2018; Huang et al., 2020). Moreover, treatment with mycelium polysaccharides from *Coprinus comatus* could attenuate the protein expression of Wnt1 and β-catenin in DN rats induced by a high-fat diet and streptozotocin (Gao et al., 2021). Sinomenine, an active alkaloid extracted from the climbing plant *Sinomenium acutum*, inhibited the expression of profibrotic proteins, including α-SMA and fibronectin, in TGF-β1-treated HEK293 human embryonic kidney cells and ameliorated tubulointerstitial fibrosis by inhibiting the protein expression of α-SMA and fibronectin in UUO mice, which was associated with the inhibition of β-catenin protein expression in TGF-β1-treated HEK293 cells (Qin et al., 2016). Collectively, our current and previous work and that of others suggest that synchronous inhibition of multiple RAS genes by blocking the hyperactive Wnt/β-catenin signalling pathway is a therapeutic strategy by which TCMs can be used to protect against renal fibrosis.

## Conclusion

In summary, our current study was the first to demonstrate that SKI could lower elevated blood pressure, improve impaired renal function and ameliorate renal fibrosis. Mechanistically, SKI could lower hypertension and abolish RAS activation by inhibiting the hyperactive Wnt/β-catenin signalling pathway. Three anthraquinones blocked multiple RAS elements by inhibiting the hyperactive Wnt/β-catenin signalling pathway. Inhibiting multiple RAS genes by blocking the activated Wnt/β-catenin signalling pathway might be considered a therapeutic strategy by which TCMs can be used to protect against renal fibrosis.

## Data Availability

The original contributions presented in the study are included in the article/supplementary materials, further inquiries can be directed to the corresponding authors.
